# Active sorting of orbital angular momentum states of light with a cascaded tunable resonator

**DOI:** 10.1038/s41377-020-0243-x

**Published:** 2020-01-28

**Authors:** Shibiao Wei, Stuart K. Earl, Jiao Lin, Shan Shan Kou, Xiao-Cong Yuan

**Affiliations:** 1 Nanophotonics Research Center, Shenzhen Key Laboratory of Micro-Scale Optical Information Technology, Shenzhen University, Shenzhen, 518060 China; 2 Department of Chemistry and Physics, La Trobe Institute for Molecular Science (LIMS), La Trobe University, Victoria, 3086 Australia; 3 School of Engineering, RMIT University, Melbourne, Victoria, 3001 Australia; 4 School of Physics, The University of Melbourne, Tin Alley, Melbourne, Victoria, 3010 Australia

**Keywords:** Applied optics, Electronics, photonics and device physics

## Abstract

The orbital angular momentum (OAM) of light has been shown to be useful in diverse fields ranging from astronomy and optical trapping to optical communications and data storage. However, one of the primary impediments preventing such applications from widespread adoption is the lack of a straightforward and dynamic method to sort incident OAM states without altering the states. Here, we report a technique that can dynamically filter individual OAM states and preserve the incident OAM states for subsequent processing. Although the working principle of this technique is based on resonance, the device operation is not limited to a particular wavelength. OAM states with different wavelengths can resonate in the resonator without any additional modulation other than changing the length of the cavity. Consequently, we are able to demonstrate a reconfigurable OAM sorter that is constructed by cascading such optical resonators. This approach does not require specially designed components and is readily amenable to integration into potential applications.

## Orbital angular momentum states: dynamic sorting

The sorting of light of different states of orbital angular momentum (OAM) is often required for applications wishing to exploit twisted light beams. Now, Shibiao Wei and coworkers from China and Australia have developed a convenient solution to the task which is reconfigurable and dynamically tunable. The scheme makes use of a Fabry Perot (FP) cavity with a tunable length that is adjusted by a piezoelectric transducer. When the length of the cavity is tuned to be resonant with the desired OAM state, it is transmitted while all the others are rejected. By cascading several such cavities and actively tuning their lengths it is possible to create a dynamically reconfigurable OAM sorter. The team tested the approach with twisted vortex light beams generated by passing 632.8 nm HeNe laser light through a spatial light modulator.

## Introduction

The optical orbital angular momentum (OAM) carried by vortex beams has been shown to be relevant to a broad range of disciplines, including quantum optics^1,2^, high-density data storage^3^, optical trapping^4^, astrophysics^5,6^, telecommunications^7–13^, high-resolution microscopy^14^, and optical interferometry for the detection of gravitational waves^15,16^. Photons entangled via OAM have also been used to demonstrate a violation of generalized Bell inequalities^17^ and for quantum cryptography^18^. Despite this broad range of applications, a method to dynamically discriminate a beam of a specific OAM state from other beams while retaining the initial state has not yet been reported in the literature.

The OAM of light is associated with the helical phase front of a propagating beam, just as spin angular momentum is connected to the circular polarization of light. *Laguerre-Gauss* (*LG*) laser modes were the first modes to be identified as carrying OAM^19^, although any beam with an azimuthal phase dependence proportional to *exp(ilθ)* has *lħ* units of OAM per photon, where the integer number *l* is known as the topological charge and *θ* is the azimuthal coordinate. A beam of the OAM state |*l* = 0〉 has a standard Gaussian intensity distribution, while a beam of any other OAM state has an intensity void (optical vortex) in its center owing to a phase singularity arising from its helical phase profile^20^.

A vortex beam of topological charge +*l* can be converted into a Gaussian beam (|*l* = 0〉) by passing the beam through an optical element that has the opposite helical phase *exp(−ilθ)*; the resultant Gaussian beam can then be coupled into a single-mode optical fiber. All other co-propagating OAM states will be unable to couple into the fiber because of the singularity at each of their centers, thus forming a simple mode-selection device^21^. A more intricate conformal transformation has been used to convert the helical phase structure of a vortex beam into a linear phase gradient using either spatial light modulators (SLMs)^22^ or custom-designed elements^23–25^. The resultant linear spread of the beam profile is able to spatially disperse OAM states based on the topological charge. Spatial dispersion of OAM states was also recently reported using a plasmonic metasurface^3^ in which nanostructured grooves in a metallic film coupled the incident OAM states to surface plasmon polaritons on a metal surface and subsequently spatially routed them to nano-ring slits based on their topological charge. Other techniques, focusing at different focal planes^26^ and on-chip sorting with plasmonics^27^, have also been reported. While spatially dispersing vortex beams through the conversion of the helical phase is an ingenious approach, it destroys the incident OAM states and is therefore more suitable for detecting the topological charge at the endpoint of an optical system. A more general sorting mechanism would be one that sends different OAM states to different output ports without altering the original states. A modified Mach-Zehnder interferometer consisting of Dove prisms and spiral phase plates has been used to construct a sorting device to select beams based on their OAM, *l*, and total angular momentum, *j* *=* *s* *+* *l*, where *s* is the spin angular momentum^1,2^. However, this method still changes the incident OAM states because it uses spiral phase plates—however, the recovery of the original states is relatively easy. In theory, an interferometric method can sort a number of OAM states. However, sorting devices using this approach require (*n* − 1) interferometers with (2*n* − 2) arms grouped at several stages to sort *n* different OAM states, which means that the complexity and losses increase rapidly with the number of OAM states to be sorted. Furthermore, a unique combination of the configurations of all interferometer arms has to be found for each given set of OAM states. The configuration at each stage of this form of interferometer network may then also need to be completely modified with the inclusion of each new OAM state. In this work, we demonstrate dynamic sorting of OAM states by using cascaded tunable resonators. By changing the resonance status of a resonator, individual OAM states are separated.

## Results

**Fig. 1:**
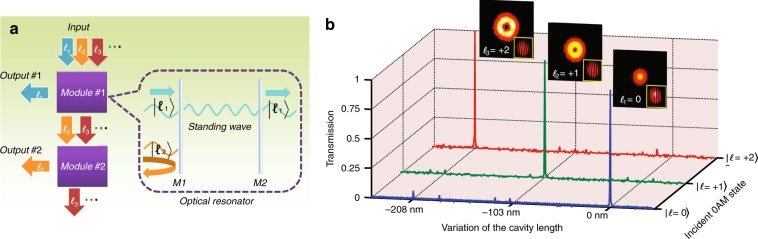
OAM sorting principle. **a** Conceptual diagram of the desired OAM sorting system based on a cascaded modular design. As an example, three different OAM states (*l*_1_, *l*_2_, and *l*_3_) enter the system. The first module outputs one state (*l*_1_) while rejecting and redirecting the other states (*l*_2_ and *l*_3_) to the next module, where state *l*_2_ is separated from the other state *l*_3_ and exits the sorter. The main component of each module here is an optical cavity/resonator tuned to a specific incident OAM state. M1 and M2 are highly reflective mirrors that form a Fabry-Pérot cavity. The resonant OAM state experiences high transmission through the cavity owing to resonant tunneling, while the non-resonant states experience destructive interference within the cavity and are therefore completely reflected. **b** Measured transmission of various OAM states as a function of the change in length of a Fabry-Pérot cavity. Experimental details are supplied in Supplementary Information Section B. The shift of the transmission peak, as measured using a photodiode, shows a clear dependence on |*l*|. Some small additional peaks correspond to parasitic cavity modes originating from imperfections in the mirrors, the slight misalignment/asymmetry of the incident beam, and the impurity within the incident OAM state. The insets show the intensity distribution of the transmission and respective fork interference pattern with a reference plane wave. The number of branches at the dislocation in the resulting interference pattern enables an identification of the value of the topological charge (|*l*|) of the OAM state, validating our claim that the transmitted beam retains its initial state

We propose a modular OAM sorting process (illustrated in Fig. 1a). In our process, each module must accept a number of input OAM states, output only one state, and divert all other states in an unaltered manner for subsequent processing. Therefore, within this modular architecture, the design of an OAM sorting system may be realized by simply cascading the modules, and the inclusion of any new OAM state can be achieved easily by connecting an additional module to the end of the chain. For the cascaded arrangement to work, it is important that each module rejects non-output OAM states with a very high efficiency so that the last output OAM state does not suffer from a significant intensity drop relative to the intensity of the first output. Mirrors are reflective optical components that can reflect (reject) incident light with almost 100% efficiency (99.9% efficiency is readily achieved by modern mirrors over the visible or infrared spectrum) while preserving most of the characteristics of the original light. However, a normal mirror cannot differentiate OAM states and acts as a barrier to all incident photons carrying different OAM states. Surprisingly, by adding another mirror (the second barrier shown in the inset of Fig. 1a), a particular OAM state can tunnel through the two barriers formed by the pair of mirrors in a fashion similar to a resonant tunneling diode. The space between the two mirrors can be considered to be an optical cavity that resonates with the selected OAM state, resulting in the tunneling of the state through the two mirrors. This configuration is, in fact, a *Fabry-Pérot* (FP) cavity^28^, a ubiquitous, high-finesse optical resonator that is used in numerous applications ranging from large-scale interferometers^29^ and resonant mode cleaning^30^ to astronomy^31^ and spectroscopy^32^. The transmission of an FP cavity can approach 100% with an extremely high resolution^33^, and the resonant properties can be manipulated by altering the cavity length, incident angle, and reflectivity of the mirrors^34,35^. Practically, most FP cavities are formed by two curved mirrors instead of parallel plane mirrors to reduce the beam walk-off and simplify the alignment of the incident beam. A propagating OAM state acquires a phase shift relative to a plane wave as it is focused by the curved mirrors. The accumulated phase shift varies with the OAM state and determines whether the state can form a standing wave in the cavity, i.e., at resonance. This phase shift is known as the Gouy phase shift and occurs as the beam propagates through the region around its focal point, which is $\left( z \right) = {\tan}^{ - 1}\left( {z/z_R} \right)$ (*z*_*R*_ is the Raleigh range of the beam). This phase shift term only depends on the cavity parameters, and this additional phase shift enables a resonant cavity to sort vortex beams of different OAM states. A resonant mode of a cavity was defined by Kogelnik as a self-consistent field configuration^34^; if a mode can be represented as a wave traveling back and forth between two mirrors, the beam parameters must by necessity be unchanged after one return trip around the cavity. This implies that in an optical cavity, resonance may only occur when the phase shift from one mirror to the other mirror is a multiple of π. An *implicit* relation between the cavity length and the topological charge of a resonant OAM state can be found (Methods) and expressed as

1 $$D = \frac{\lambda }{2}\left[ {q + \left( {\left| l \right| + 1} \right)\frac{\varphi }{{\it{\uppi }}}} \right]$$

where *D* is the cavity length; $\varphi = \arccos \left( { \pm \sqrt {\left( {1 - D/R_1} \right)\left( {1 - D/R_2} \right)} } \right)$ is the accumulated Gouy phase shift experienced by the light traveling from one end of the cavity to the other, which is determined by the mirror curvatures (*R*_1_ and *R*_2_); *λ* is the wavelength of light; and *q* is an integer. Since the resonance is sensitive to even subwavelength movements (e.g., a few hundred nanometers), the transcendental equation can be approximated by an *explicit* relation between a small variation ∆ in the cavity length and the resonant topological charge (Supplementary Information Section A):

2 $$\Delta = \frac{\lambda }{2}\left[ {q + \left( {\left| l \right|} \right)\frac{\varphi }{{\it{\uppi }}}} \right]$$

Hence, one can resonantly match a specific OAM state to an FP cavity by tuning the cavity length, producing an OAM filter that allows the transmission of a selected state and the rejection of all others. The resonant filter then serves as the key component in the constituent module of an OAM sorting system.

A scanning FP cavity whose length can be actively tuned using a piezoelectric transducer was selected for the sorting experiment. A liquid-crystal-based spatial light modulator (SLM) was used to prepare various incident OAM states^36^.

Because an FP cavity with a high finesse is highly sensitive to wavelength instabilities and the broad spectrum of the OAM states, a frequency-stabilized HeNe laser with a wavelength of 632.8 nm is used. A frequency instability of the laser will lead to adrift of the resonant peaks. In an actual application, decreasing the finesse of the cavity will widen the resonant peak, increasing the tolerance to peak drift but limiting the number of OAM states that the system can distinguish. Therefore, there should be a trade-off between the performance of the laser beam and the finesse of the cavities in actual applications.

Figure 1b clearly shows that a sharp transmission peak occurs at different cavity lengths for each OAM state. In other words, the FP cavity acts as a filter, transmitting a specific OAM state for every correctly selected cavity length. At resonance, we also verified that the transmitted vortex beam remains in its original OAM state. These experimental results are corroborated by numerical calculations performed using FINESSE^37^, an interferometer simulation package. The (absolute) cavity length used in these simulations was calculated using the change in the cavity length relative to the original resonated cavity length of a Gaussian beam using the *l*-dependent Gouy phase accumulated by a vortex beam within the FP cavity (see Supplementary Information Section C).

### Separation of co-propagating OAM states

**Fig. 2:**
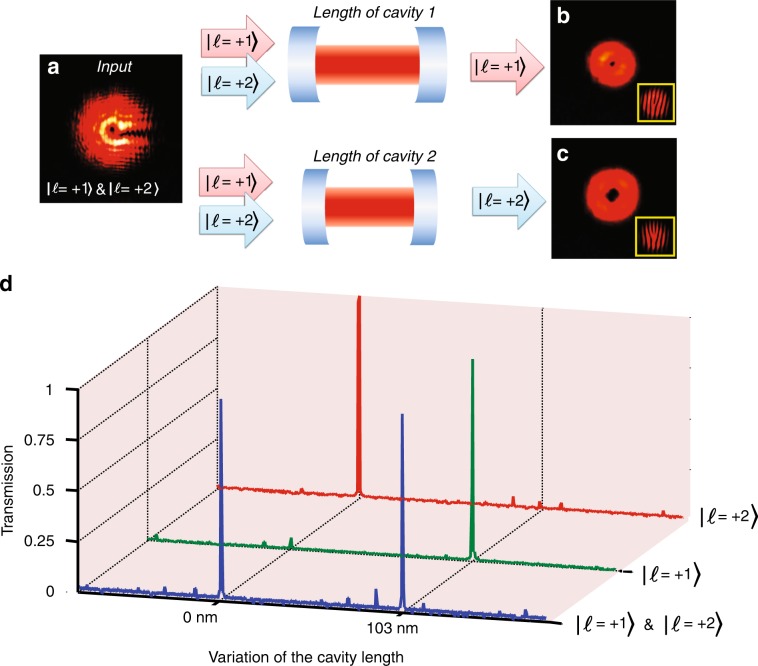
Separation of co-propagating OAM states. **a** Intensity distribution of the incident beam with the superposition of two OAM states (|*l* *=* *+1〉* and |*l* *=* *+2〉*). **b**, **c** Transmitted intensity distributions after the FP cavity is tuned on resonance. The topological charge of the transmitted OAM state is quantified via the fork-like interference patterns **d**, **e**. **f**. Transmission of the superposition OAM states as a function of the cavity length (blue line), accompanied by reference spectra of the two individual OAM states (green: |*l* *=* *+1〉*; red: |*l* *=* *+2〉*). The much smaller peaks in the data mainly originate from errors inherent in the iterative algorithm used to generate the superimposed states

As an essential part of an OAM sorter, the FP cavity must be able to isolate a specific OAM state from a number of co-propagating states. Figure 2 shows that the FP cavity dynamically disperses the input beam, transmitting each constituent OAM state at different cavity lengths. To prepare a laser beam carrying two distinct OAM states (|*l* *=* *+1〉* and |*l* *=* *+2〉*) using a single SLM, an iterative algorithm was used to create the appropriate phase pattern^38^, which was then displayed on the SLM to convert an incident Gaussian beam into the superposition of two vortex states used here. By tuning the cavity length, different OAM states were selected. To intuitively detect the topological charges of the selected OAM states, the interference patterns between the OAM beam and a plane wave with a tilt incident angle were obtained, as shown in Fig. 2d, e. Two distinctive transmission peaks occur at the same cavity lengths as those of the reference beams carrying a single OAM state, as shown in Fig. 2f. The much smaller peaks in the data primarily originate from errors inherent in the iterative algorithm used to generate the superimposed states. The temperature variation and the displacement gaps between the increasing voltage and the decreasing voltage will influence the accuracy of the measurement of the cavity length. The interval between the states (|*l* *=* *+1〉* and |*l* *=* *+2〉*) was 103 nm, which had a 2 nm shift compared with the value in Fig. 1.

### Modular design of an OAM sorter

**Fig. 3:**
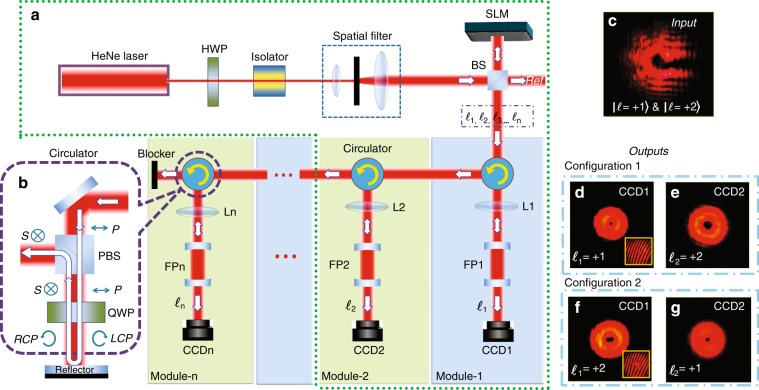
Modular design of an OAM sorter. **a** Experimental setup of the OAM sorter. A half-wave plate (HWP) is used to rotate the linear polarization axis of the laser beam prior to spatial filtering to facilitate the alignment with the optical axis of the SLM. An isolator is used to prevent unwanted reflected light from entering the frequency-stabilized laser cavity. The superimposed OAM states are prepared by an SLM loaded with specially designed patterns. A reference beam is generated from one of the ports of a non-polarizing beam-splitter (BS) for interference with the output OAM states in the latter part of the experiment. A lens (L1) is used to focus the incident beam to the first cavity (FP1) with the size of the focal spot matched to the cavity mode. The transmitted signal from each FP cavity is monitored using either a photodiode or a CCD camera. While only two FP cavities (enclosed in the dashed box) are used in this experiment, the high sensitivity and signal-to-noise ratio of the cavities allow a number of cavities to be cascaded to maximize the number of OAM states being sorted. **b** Optical circulator consisting of a polarizing beam-splitter (PBS) and a quarter-wave plate (QWP). The incident light is p-polarized (linear polarization). Light reflected from the FP cavity passes the QWP twice, transforms into the orthogonal s-polarization state, and therefore exits the PBS from a different port. RCP: right-handed circular polarization; LCP: left-handed circular polarization. **c**–**g** Intensity distributions and fork-like interference patterns when the pair of FP cavities are each transmitting a different OAM state. These images were taken at the two output ports when both cavities were tuned on resonance for one of the two OAM states. To capture these images, the first cavity was tuned on resonance to transmit |*l* = +*1〉* and the second cavity was tuned to transmit |*l* *=* *+* *2〉* (and vice versa). We have also shown that the output OAM states can be swapped between the two ports dynamically by varying the cavity lengths (Configuration 1 vs. Configuration 2)

For the off-resonant OAM states, the FP cavity consists simply of two mirrors with very high reflectances. The high reflectivity, coupled with the fact that the cavity preserves all incident OAM states (for both transmission and reflection), enables the implementation of a modular design in which multiple cavities are cascaded, allowing simultaneous sorting of multiple OAM states. To demonstrate this implementation, a second FP cavity is added to construct an OAM sorter with two output ports, as shown in Fig. 3. An optical circulator^37^ is used to direct the reflected states from the first cavity to the second cavity. We show that it is possible to separate OAM states using cascaded cavities, enabling the simultaneous sorting/detection of multiple co-propagating OAM states within a single beam. Furthermore, the path taken by a particular OAM state within the sorter can be manipulated in real time by altering the cavity lengths of the modules, permitting dynamic routing of an OAM state to any output port. Since the resonant cavity length only depends on the modulus of the topological charge, as indicated by Eq. 1, two OAM states with topological charges of opposite sign are degenerate and cannot be differentiated using a single FP cavity. One can choose to either use only positive (or negative) topological charges in the system or add spiral phase plates and a second FP cavity to the module to remove the degeneracy (Supplementary Information Section D).

### Simultaneous sorting of OAM and wavelength

FP cavities are regularly used to differentiate finely spaced laser lines. This ability complements our approach to sorting OAM states by permitting simultaneous differentiation of optical signals based on both wavelength and the OAM state within the same device. Here we demonstrate simultaneous sorting of wavelengths and OAM states using a single FP cavity.

**Fig. 4:**
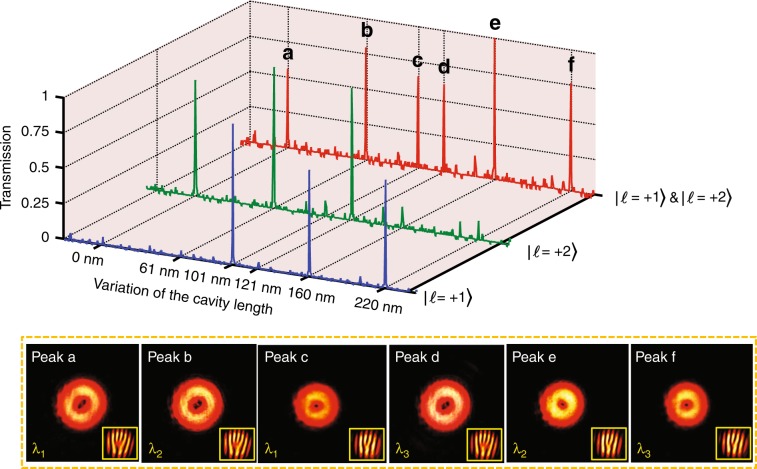
Simultaneous sorting of OAM and wavelength. Simultaneous OAM and wavelength multiplexing/demultiplexing in an incident beam. The blue and green lines indicate the single OAM states generated from the same laser with three laser lines (the central wavelength is 632.816 nm in air, and the separation of each laser line is 0.000584 nm), while the red line represents an incident beam with both OAM states at the three laser lines. The peaks of the mixed OAM states match those of the individual OAM states. Insets: CCD images of each of the six resonant transmissions

The frequency-stabilized HeNe laser used in the previous sections of this paper was replaced with another HeNe laser that is known to lase on three longitudinal modes (Melles Griot 05-LHR-151, with a mode spacing of 438 MHz, producing three laser lines with a separation of approximately 0.000584 nm) centered at 632.816 nm in air. The remainder of the experimental setup remained unchanged, as shown in Fig. 1. The three laser lines, which would be indistinguishable using a diffraction grating, are clearly visible in Fig. 4 owing to the superior resolution of the FP cavity.

An input superposition of two OAM states (|*l* = +1〉 and |*l* = +2〉) across the three wavelengths of the laser was directed through the FP cavity. The results of this experiment are shown in Fig. 4 (red line) with additional results from the single OAM state for reference. The six transmission peaks account for all possible combinations of the two OAM states and the three wavelengths. These results show that the OAM sorter can be used to separate wavelengths simultaneously without requiring additional components.

## Discussion

In conclusion, we have demonstrated a reconfigurable OAM sorter that handles multiple OAM states simultaneously while preserving the original states. In addition, with this modular design, the system can be easily scaled with an increasing number of OAM states. The basic building element of this OAM sorter is an optical cavity; thus, the sorter inherits a number of merits from using a resonance-based approach, such as high selectivity and high efficiency. The cross-talk between the various OAM states is suppressed because of the resonant nature of this approach. Furthermore, by harnessing the principle of Gouy phase accumulation, the sorter is sensitive not only to the values of the azimuthal mode index *l* but also to the radial mode index *p*. Compared with an interferometer that was previously proposed for sorting the radial modes of *LG* beams^39^, the sorter proposed here is more compact, which is important when considering actual applications. The transmission of the resonant OAM states is experimentally measured to be approximately 92% of the maximum efficiency (21.7%) of the particular off-the-shelf FP cavity used in the experiment. While FP cavities can theoretically be 100% efficient, in practice, they are limited by the surface roughness of the mirror coatings/surfaces, alignment errors, and other imperfections. For a high-finesse cavity, such as those used in cavity quantum electrodynamics (QED) experiments, scattering and absorption losses from the mirrors are the major sources of loss^40^. Moving from the visible region (~632.8 nm) to the infrared region to minimize surface scattering and material absorption losses is the simplest way to improve the efficiency. An optimization of the FP cavity and the use of dielectric mirrors would also increase the efficiency. For example, the transmission efficiency could be significantly improved with a high-efficiency cavity and a longer working wavelength; for example, over 99% transmission efficiency has been reported experimentally using mirrors with a higher reflectance in the cavity and by moving to longer wavelengths such as 1064 nm (Supplementary Information Section E)^41^). Furthermore, because an FP cavity with a high finesse is highly sensitive to wavelength instabilities and the broad spectrum of the OAM states, a frequency-stabilized HeNe laser with a wavelength of 632.8 nm is used in the experiment. A frequency instability of the laser will lead to a drift of the resonant peaks. In an actual application, decreasing the finesse of the cavity will widen the resonant peak, thus increasing the tolerance to peak drift but also limiting the number of OAM states that the system can distinguish. This trade-off between the performance of the laser beam and the finesse of the cavities will therefore need to be considered in light of specific real-world applications. Furthermore, we have reported an OAM healing effect by using an FP cavity^42^. An obstructed OAM beam can be healed when passing through an FP cavity, which will greatly increase the stability of the whole OAM application system.

In conjunction with wavelength and polarization, mode-division multiplexing (MDM) using optical OAM states has been investigated because of the infinite number of orthogonal states potentially available for increasing the transmission capacity of a network. OAM-enabled optical networks have already demonstrated capacities greater than 1 Terabit per second^7,8,13^. While increases in the data-carrying capacity of single-mode fibers have outweighed the demand over the course of the previous three decades, our ever-expanding demand for more data is predicted to exceed the current capacities within a decade^43^. The dynamic nature of the OAM sorter offers a simple avenue for constructing reconfigurable optical networks to address this shortfall from another perspective. The mature nature of optical cavity technology, the high quality of available materials, and the ability to reduce the cavity dimensions down to on-chip and in-fiber cavities all indicate that this approach could have a wide-reaching impact. Our OAM sorting technique may also be of interest to imaging, as hyperspectral imaging camera arrays now integrate FP cavities on a single-pixel level^44^, and quantum optics researchers may find interesting implications in these findings for cavity QED experiments^45,46^.

## Materials and methods

The mechanism behind the OAM-sorting ability of a Fabry-Pérot (FP) cavity is the Gouy phase shift that a resonant beam accumulates within the cavity. This phenomenon, in which a propagating wave acquires a phase shift relative to a (theoretical) plane wave as it is focused by an optical system, was first observed in 1890^47^.

**Fig. 5:**
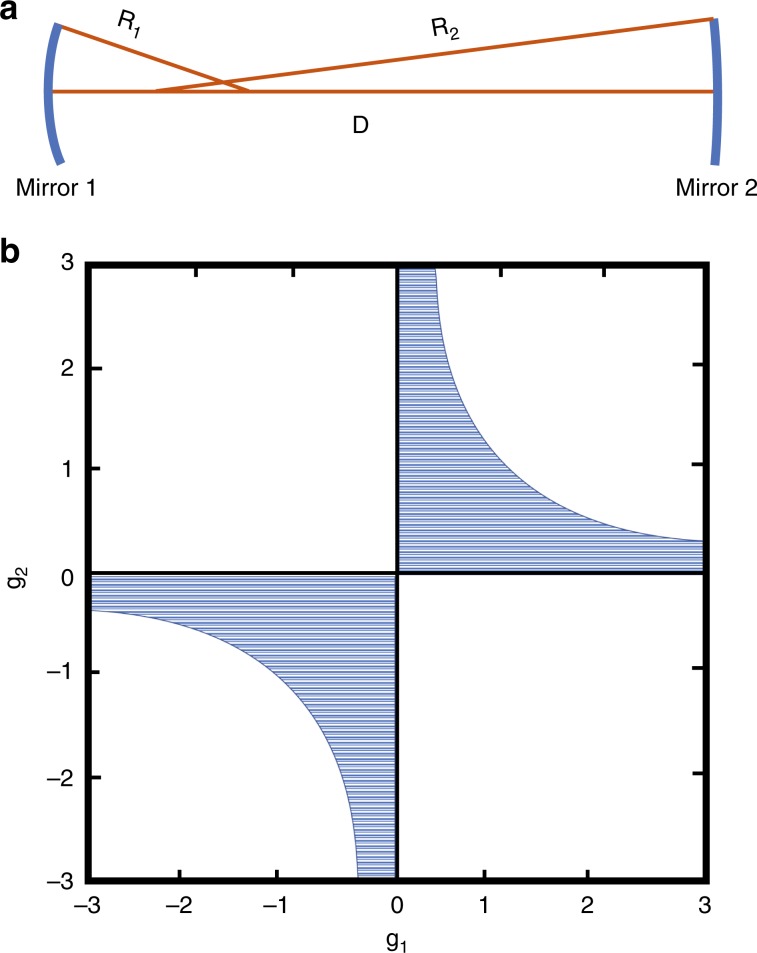
**a** Schematic of a generic two-mirror Fabry-Pérot cavity comprising two concave mirrors. The radii of curvature are marked in the figure. **b** Stability diagram of the optical cavity. The shaded regions indicate the stable cavity regions. The hyperbola bounding the shaded regions represents *g*_1_*g*_2_ = 1

Figure 5a shows a schematic of a generic two-mirror optical cavity comprising concave mirrors with different radii of curvature. The stability of such a cavity can be calculated by ray transfer analysis^34^. The result of this analysis commonly introduces the *g* factors of a cavity, defined as

3 $$g_{1,2} = 1 - D/R_{1,2}$$

where *R*_*1,2*_ is the radius of curvature of the specified mirror and *D* is the mirror separation, such that a cavity is stable provided that

4 $$0 \le g_1g_2 \le 1$$

A stable cavity is one in which a paraxial ray injected into the resonator does not escape but rather remains confined near the longitudinal axis of the cavity. In contrast, an unstable cavity will allow light, even on-axis light, to eventually escape. Fig. 5b illustrates the combinations of the *g* factors that result in a stable cavity in the shaded regions. The commercial scanning FP cavity used in our experiment was originally confocal (*D* *=* *R*_*1*_ *=* *R*_*2*_), corresponding to the origin (0,0) in the above diagram. The advantage of a confocal FP cavity is that all longitudinal and transverse modes occur at the same cavity length. Since this experiment relied on a cavity to disperse the input spatial modes, the cavity length was extended (*R*_*1*_ *=* *R*_*2*_ *=* *R* *<* *D* *<* *2* *R*) so that the cavity was no longer the case. The resultant cavity resides in the lower-left quadrant of the stability diagram owing to the convention of concave mirrors having a positive radius of curvature in this context.

To study the detailed field distributions within the FP cavity, we chose Laguerre-Gauss (LG) modes, a family of solutions to the paraxial wave equation in cylindrical coordinates, which, as discussed, were previously shown to carry OAM^19^. The general solutions are of the form

5 $$u_{pl}\left( {r,\theta ,z} \right) = \frac{C}{{\sqrt {1 + z^2/z_R^2} }}\left( {\frac{{r\sqrt 2 }}{{w\left( z \right)}}} \right)^lL_p^l\left( {\frac{{2r^2}}{{w\left( z \right)^2}}} \right) \times exp\left( {\frac{{ - r^2}}{{w\left( z \right)^2}}} \right)exp\left( {\frac{{ - ikr^2z}}{{2\left( {z^2 + z_R^2} \right)}}} \right) \times exp\left( { - il\theta } \right)exp\left( {i\left( {2p + |l| + 1} \right)\psi \left( z \right)} \right)$$

In this equation, *u*_*pl*_ is the eigensolution to the paraxial wave equation; *C* is a normalization constant; *L*_*p*_^*l*^ is the Laguerre-Gauss polynomial of order (*p,l*); *p* and *l* are the radial and azimuthal mode indices, respectively; *w(z)* is the standard definition of the beam waist; *r*, *θ*, and *z* are the radial, azimuthal and longitudinal coordinates, respectively; and $z_R$ is the Raleigh range of the beam, defined as

6 $$z_R = \frac{{\pi w_0^2}}{\lambda }$$

Here, *w*_0_ is the standard definition of the beam waist. The Raleigh range is the distance the beam needs to propagate from the waist for the width of the beam to increase by √2, which corresponds to an on-axis intensity of half the peak intensity at that point.

The term $\psi \left( z \right) = {\tan}^{ - 1}\left( {z/z_R} \right)$ represents the Gouy phase shift as the beam propagates through the region around its focal point. The above equation shows that for higher-order *LG* modes, there is an additional Gouy phase shift relative to a Gaussian beam owing to the additional transverse structure of the beam. This additional phase shift enables a resonant cavity to sort vortex beams of different OAM states.

A resonant mode of a cavity was defined by Kogelnik as a self-consistent field configuration^34^; if a mode can be represented as a wave traveling back and forth between two mirrors, the beam parameters must by necessity be unchanged after one return trip around the cavity. This implies that in an optical cavity, resonance may only occur when the phase shift from one mirror to the other mirror is a multiple of π.

The total phase accumulated by an *LG* beam traveling from one side of a stable optical cavity to the other side (i.e., mirror 1 at *z*_1_ and mirror 2 at *z*_2_) can be written as

7 $$\emptyset \left( {z_2 - z_1} \right) = kD - \left( {2p + |l| + 1} \right)\left[ {\psi \left( {z_2} \right) - \psi \left( {z_1} \right)} \right]$$

The first term on the right-hand side represents the phase advance caused by light with a propagation constant *k* traveling a distance *D*, while the second term represents the accumulated Gouy phase within the cavity. This second term was shown by Siegman to depend only on the cavity parameters^33^, i.e.,

8 $$\psi \left( {z_2} \right) - \psi \left( {z_1} \right) = \varphi = arccos\left( { \pm \sqrt {g_1g_2} } \right)$$

where the *g* factors are as defined previously. The choice of sign in the definition of the Gouy phase depends on the position of the cavity in the stability diagram (Fig. 5b). The “+” sign applies to the upper-right quadrant, while the “−” sign applies to the lower-left quadrant.

Based on the previous equation and the knowledge that the condition for a standing wave is that the total round trip (total distance traveled of 2*D*) phase must be an integer multiple of 2π, a mode must satisfy the following equation to be resonant with the cavity:

9 $$\frac{{\omega 2D}}{c} - 2\left( {2p + |l| + 1} \right)\varphi = 2q\pi$$

where *q* is an integer. Here, the substitution of $k = \frac{\omega }{c}$ was used to facilitate the derivation of the resultant resonance frequencies of the longitudinal-plus-transverse modes of the cavity, i.e.,

10 $$\omega = \omega _{qpl} = \frac{{\pi c}}{D}\left( {q + \frac{{\left( {2p + |l| + 1} \right)}}{\pi }\varphi } \right)$$

where the factors of 2 have been canceled. This equation tells us that for a cavity with a fixed length *D*, different *LG* modes will resonate with the fixed cavity at slightly different frequencies because of the influence of the Gouy phase.

For a dynamic cavity such as the scanning FP cavity used in this experiment, it is possible to rearrange the previous equation, as *D* is variable while the frequency is fixed. The result is that for an *LG*^*l*^_*p*_ mode of wavelength λ within a cavity, the resonant cavity length, *D*, is

11 $$D = \frac{\lambda }{2}\left\{ {q + \left( {2p + \left| l \right| + 1} \right)\frac{\varphi }{\pi }} \right\}$$

Here, $\lambda = 2\pi c/\omega$ was used to simplify the resulting equation. This equation shows that owing to the accumulated Gouy phase shift experienced by a beam traveling from one mirror to the other mirror, the resonant length of the cavity depends on the values of the azimuthal mode index (OAM topological charge) and the radial mode index. Since we are interested in the sorting of OAM (the azimuthal mode index *l*), the radial mode index *p* is irrelevant in this case and thus is set to zero to further simplify the equation:

12 $$D = \frac{\lambda }{2}\left\{ {q + \left( {\left| l \right| + 1} \right)\frac{\varphi }{\pi }} \right\}$$

This dependence is the mechanism that enables the FP cavity to differentiate vortex beams of different OAM states. As the cavity length is varied, different OAM states resonate within the cavity (and are transmitted), while all other states experience destructive interference (and are therefore reflected).

## Supplementary information

Supplementary Information for Active sorting of orbital angular momentum states of light with a cascaded tunable resonator
