# Supplementary material for: Active sorting of orbital angular momentum states of light with a cascaded tunable resonator
Source: Light Sci Appl. 2020 Jan 28;9:10. doi: 10.1038/s41377-020-0243-x (PMC6987156; doi:10.1038/s41377-020-0243-x)
Supplement: Supplementary file 1 — Supplementary Information for Active sorting of orbital angular momentum states of light with a cascaded tunable resonator [file 41377_2020_243_MOESM1_ESM.docx]

Supplementary Information for ‘Active sorting of orbital angular momentum states of light with a cascaded tunable resonator’

Shibiao Wei^1–3,†^, Stuart K. Earl^2,3,†^, Jiao Lin^1,3,4,*^, Shan Shan Kou^2,*^, and Xiao-Cong Yuan^1,*^

*^1^Nanophotonics Research Center, Shenzhen Key Laboratory of Micro-Scale Optical Information Technology, Shenzhen University, Shenzhen 518060, China*

^2^Department of Chemistry and Physics, La Trobe Institute for Molecular Science (LIMS), La Trobe University, Victoria 3086, Australia.

^3^School of Engineering, RMIT University, Melbourne, Victoria 3001, Australia.

^4^School of Physics, The University of Melbourne, Tin Alley, Melbourne, Victoria 3010, Australia.

*Correspondence to: jiao.lin@unimelb.edu.au, s.kou@latrobe.edu.au, or xcyuan@szu.edu.cn

†These authors contributed equally to this work.

The Supplementary Information includes numerical calculations that support our findings, experimental details, and a discussion on the accumulated Gouy phase of a beam within an optical cavity.

**Section A—Linear relation between the variation of the cavity length and the topological charge of the resonant OAM state**

We begin by expanding Equation (1) in the main text and define *D* within this section as the resonant length of the cavity for a Gaussian beam (*p* = *l* = 0), i.e.

$D=\frac{\lambda}{2} \left\{ q+\frac{\varphi}{\pi} \right\}=\frac{\lambda}{2} \left\{ q+\frac{\arccos\left( \pm\sqrt{g_{1}g_{2}} \right)}{\pi} \right\}=\frac{\lambda}{2} \{q+\frac{arccos(\pm\sqrt{\left( 1-\frac{D}{R_{1}} \right)\left( 1-\frac{D}{R_{2}} \right)})}{\pi}\}$. (S1)

By fixing *D* as the initial cavity length, the change in cavity length for other *LG* modes becomes a correction (Δ) to that length. The equation for cavity length as a function of the radial and azimuthal mode index then becomes

$D+\Delta=\frac{\lambda}{2} \{q'+\left( 2p+\left| l \right|+1 \right)\frac{arccos(\pm\sqrt{\left( 1-\frac{\left( D+\Delta\right)}{R_{1}} \right)\left( 1-\frac{\left( D+\Delta\right)}{R_{2}} \right)})}{\pi}\}$.

(S2)

We can substitute the definition for *D* using equation (S1), and, after collecting like terms, arrive at

$\Delta=\frac{\lambda}{2}\left\{ \left( q^{'}-q \right)+\frac{2p+\left| l \right|}{\pi}arccos\left( \pm\sqrt{\left( 1-\frac{D}{R_{1}} \right)\left( 1-\frac{D}{R_{2}} \right)} \right) \right\}$. (S3)

Here the approximation is made that *(D+Δ)/R*_1,2_ *≈ D/R*_1,2_. This is a valid assumption because Δ is on the order of a fraction of a wavelength and thus approximately five orders of magnitude smaller than both *D* and *R*.

By taking $q^{''}=q^{'}-q$, $p=0$, and $\varphi=arccos\left( \pm\sqrt{g_{1}g_{2}} \right)$, we arrive at the linear relation:

$\Delta= \frac{\lambda}{2}\left\{ q''+|l|\frac{\varphi}{\pi} \right\}$. (S4)

In relation to the experiment under discussion here there are two further simplifications that can be made. The two cavity mirrors are identical, having radii of curvature, *R*, equal to 50 mm each, which simplifies the argument of the final term. One consideration of the experimental technique requires that an additional assumption is made. Because the change in the resonant cavity length is measured relative to that of the same Gaussian transmission peak each time, the implicit assumption has been made that *q’ = q*. Were this assumption not made, the cavity length change, Δ, would need to be referenced to the *nearest* transmission peak of a Gaussian beam, complicating both the data collection and analysis. With these simplifications the relevant equation here becomes

$\Delta=\frac{\lambda}{2}\frac{\left| l \right|}{\pi}arccos\left( \pm\left| 1-\frac{D}{R} \right| \right)=\frac{\lambda}{2}\frac{\left| l \right|}{\pi}arccos\left( \frac{D}{R}-1 \right)$. (S5)

As a final step, the term on the right-most side of the equation emerges when the negative sign of the argument in brackets is taken. This selection is made in light of the fact that both *g*-parameters are negative and considering where on the stability diagram (Section A) the cavity in question sits. The stability condition demands the argument be between zero and one for a stable cavity to exist, and so the modulus sign is ignored here.

The linear dependence on the resonant topological charge was further verified using the experimental data. Figure S1 shows these data (blue crosses) for the first four resonantly transmitted OAM states. The red line is a linear fit using the above equation performed using the curve fitting toolbox provided by MATLAB. The inferred cavity length using the slope of the line was found to be 70.60 mm, which agrees with our expectations based on the width of the components used to modify the FP cavity.


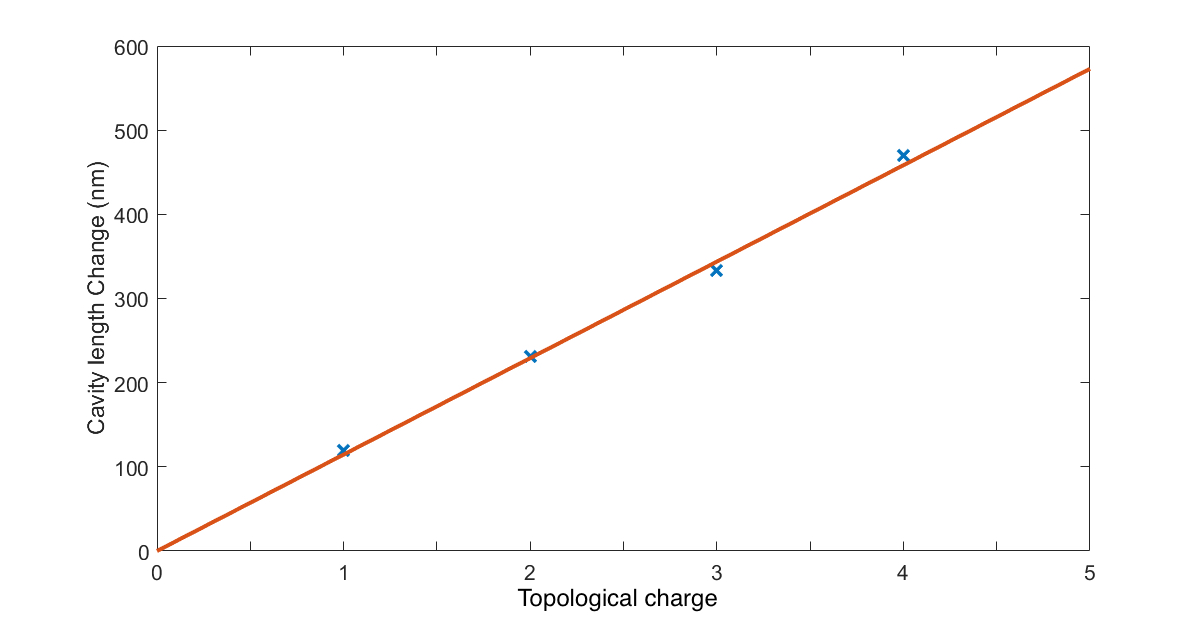


Figure S1: Variation of the cavity length as a function of the resonant topological charge. Crosses represent experimental data, while the line is a fit using Equation (S5).

**Section B—Experimental details**

Spatial light modulators (SLMs) are commonly used to produce beams of light carrying OAM. As shown in Figure S2, an SLM (Boulder Nonlinear Systems, pixel pitch 15 μm × 15 μm, fill factor 83.4%) was used to reflect an incident, linearly polarized beam from a collimated, frequency-stabilized HeNe laser (Thorlabs HRS015B, vacuum wavelength 632.991 nm). The output from the laser was sent through an optical isolator and spatially filtered using a 15-μm-diameter pinhole before being re-collimated to produce a clean Gaussian beam. The re-collimated beam was then reflected off the surface of the SLM to produce the desired OAM state. The SLM was set to display a phase-only interference pattern between a plane wave and a vortex beam of the desired topological charge.

The resulting beam was aligned to a scanning FP cavity (a modified Thorlabs SA200-5B, minimum finesse 250, original free spectral range (FSR) of 1.5 GHz, driven by an SA-201 piezo controller) with the output monitored by a CCD camera or a photodiode. Size matching of a Gaussian beam to the cavity was performed using a thin lens of focal length 250 mm. Only small corrections were required to match the vortex beams to the cavity following this initial alignment. The transmitted signal was monitored via the output of a photodiode as a function of cavity length, which greatly facilitated fine mode-matching adjustments.


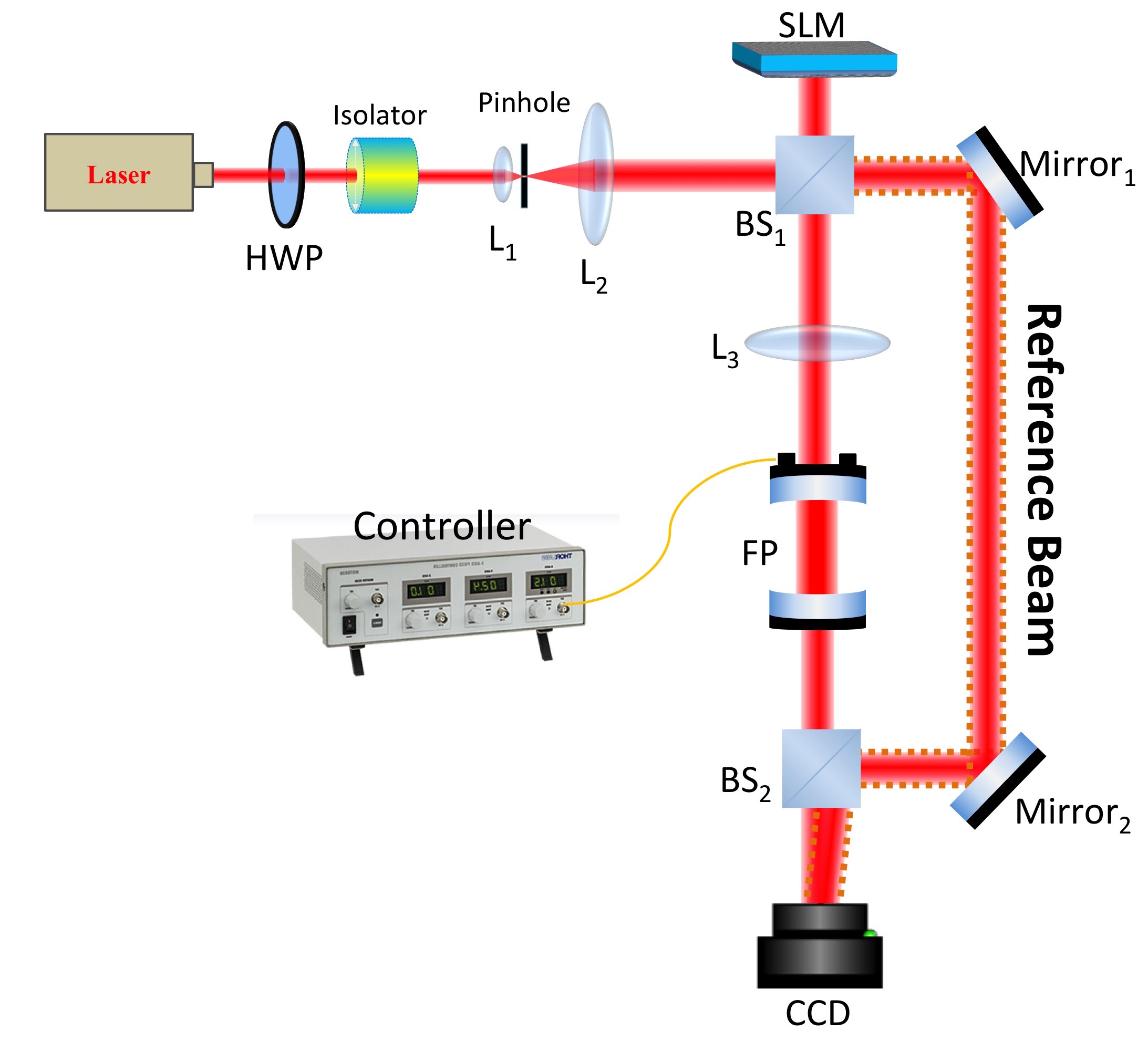


Figure S2: Illustration of the experimental setup. A linearly polarized beam of a stabilized HeNe laser is sent through a half-wave plate and an optical isolator before being spatially filtered using a 15-μm-diameter pinhole. The half-wave plate was used to rotate the orientation of the (linear) polarization axis of the beam. A telescope was incorporated into the spatial filtering of the beam (focal length of L_1_ (L_2_) was 11 mm (25.4 mm) to expand the spot size to facilitate mode matching to the cavity. The filtered beam then travelled through a 50/50 beam splitter (BS_1_), which allowed for normal incidence onto the SLM while also providing an additional reference Gaussian beam to form the forked interference patterns. The beam was reflected off of the SLM and returned through the beam splitter and mode-matched to the FP cavity using a thin lens with a focal length of 250 mm. The cavity length was controlled using a matched Thorlabs piezo controller, while the transmitted signal was monitored using either a CCD camera or photodiode.

To measure the topological charge of the transmitted OAM state, a non-polarizing beam splitter was used prior to the SLM to sample part of the initial Gaussian beam. Two mirrors were used to redirect this beam to interfere with the transmitted beam at a slight angle to the CCD camera. An alternate piezo controller (Thorlabs MDT694B) was used to maintain the cavity length while imaging the transmitted beam.

It is important to highlight that the commercial scanning FPs used throughout these experiments were modified from their off-the-shelf configuration. The Thorlabs SA200-5B FP cavities are equipped with high reflectivity (~99.375%) mirrors with 50 mm radii of curvature in a confocal configuration. This configuration results in a complete degeneracy of the longitudinal and transverse cavity modes, and thus they transmit all resonant modes at identical cavity lengths. By adding a small extension to one mirror of around 20 mm the degeneracy of the modified FP is broken.

**Section C—Simulations of the resonant modes inside a FP cavity**

Numerical calculations were performed using FINESSE (Frequency domain Interferometer Simulation SoftwarE)^1^ to confirm our experimental findings.

Figure S3 compares a FINESSE simulation with the experimental data as a function of the cavity length for different resonant OAM states. The two plots share the same legend for simplicity. The upper plot is the output of FINESSE while the lower plot is representative experimental data from a single cavity.

These numerical and experimental data present a qualitative match and serve to confirm our findings regarding the sensitivity of a FP cavity to OAM states.


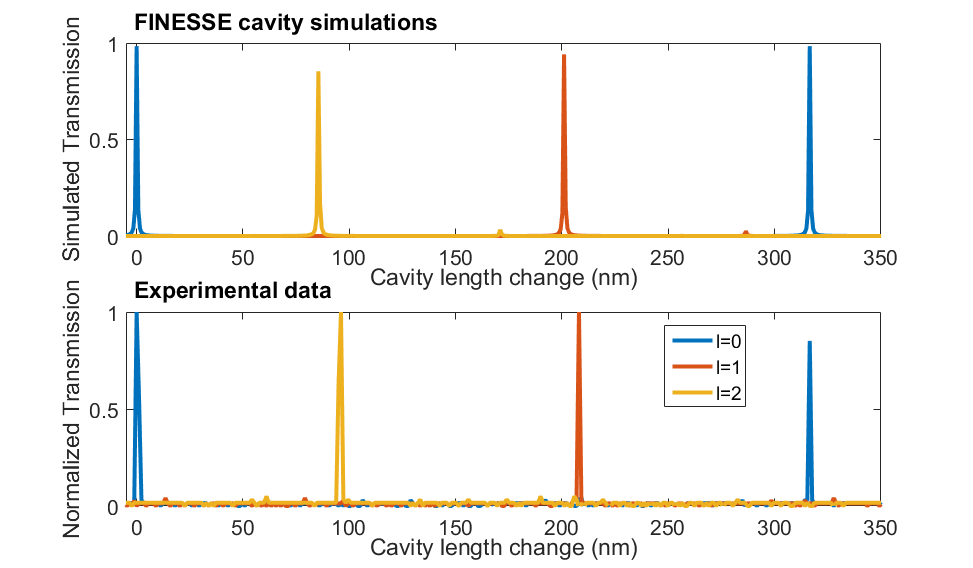


Figure S3: (top) FINESSE simulations and (bottom) experimental data as a function of the length variation of a FP cavity for three different OAM states.

**Section D—Breaking the sign degeneracy of the OAM states**

The main panel of Figure S4 shows the transmission of the scanning FP cavity for two incident beams of a pure OAM state |*l=+1⟩* and of the superposition of |*l=+1⟩* and |*l=−1⟩* in blue and green, respectively. Clearly, the system is unable to differentiate the two incident signals carrying opposite OAM states as the blue and green transmission peaks occur for the same cavity length. The red line shows the reference transmission of an incident beam of the superposition of |*l=0⟩* and |*l=+2⟩* launched via the appropriate display on the SLM. The cyan line, which shows a high level of similarity to the reference transmission, was initially a superposition of |*l=+1⟩* and |*l=−1⟩* that was sent through a spiral phase plate (SPP, *n=+1*, Holo/Or Ltd. Overall efficiency: 95%), converting it to a superposition of |*l=0⟩* and |*l=+2⟩*. The initially indistinguishable opposite OAM states can now be sorted in a fashion similar to other OAM states. Hence, a more intricate design consisting of two FP cavities as illustrated in Figure S5 can be used to replace the constituent module of an OAM sorter for breaking the sign degeneracy.


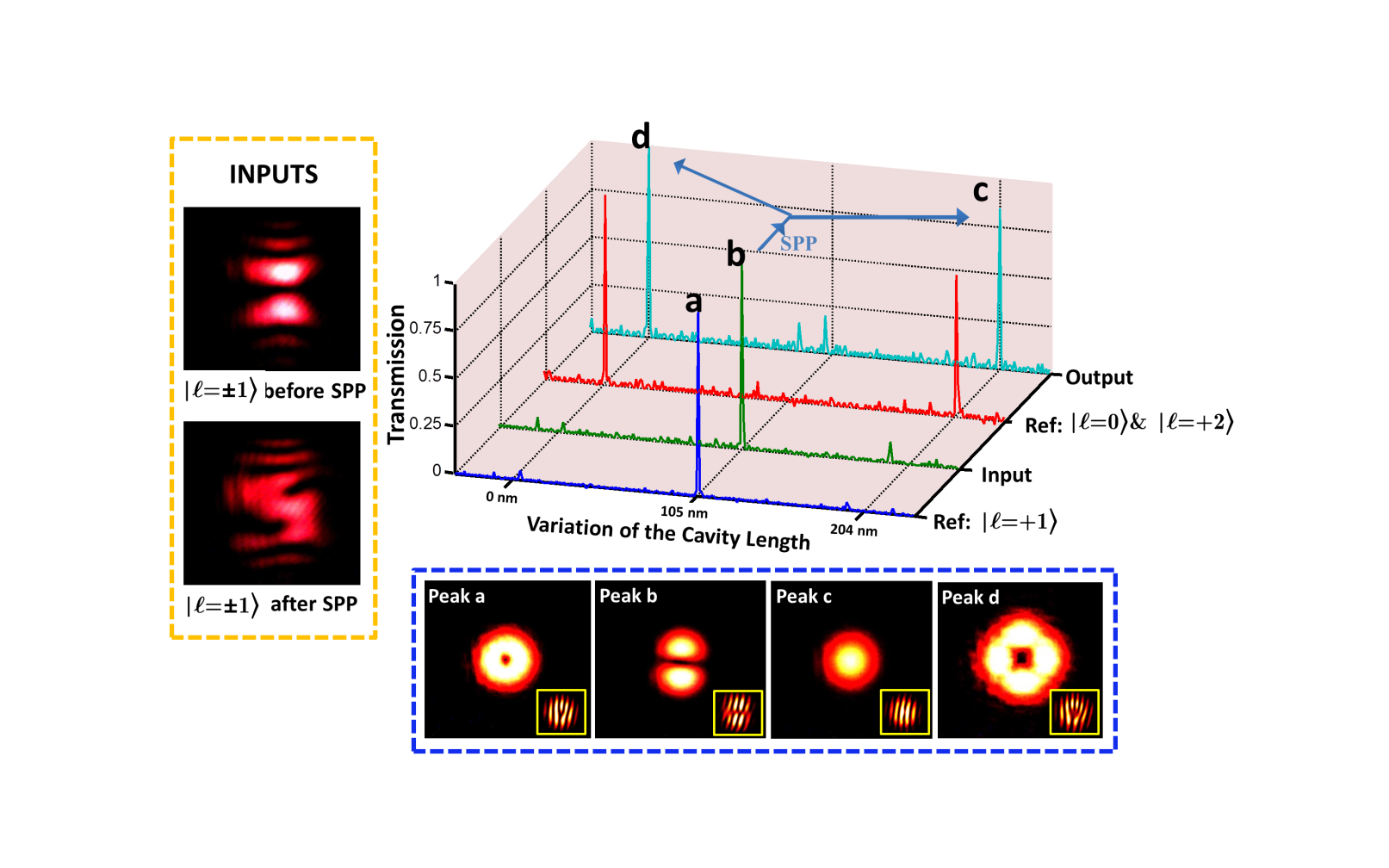


*Figure S4: Breaking the degeneracy of two OAM states with opposite signs. (main) Comparison of transmissions of single OAM state (l=+1, in blue), sign-degenerate OAM states (l=+1* *and l=−1, in green), reference SLM-created superimposed states (l=0 and l=+2, in red), and the post-SPP degenerate pair (in cyan). The inset at the bottom shows the intensity distributions of the transmission peak labeled by letters a–d in the main figure. The panel inset on the left shows the intensity distribution of the degenerate pair before (top) and after (bottom) the SPP.*


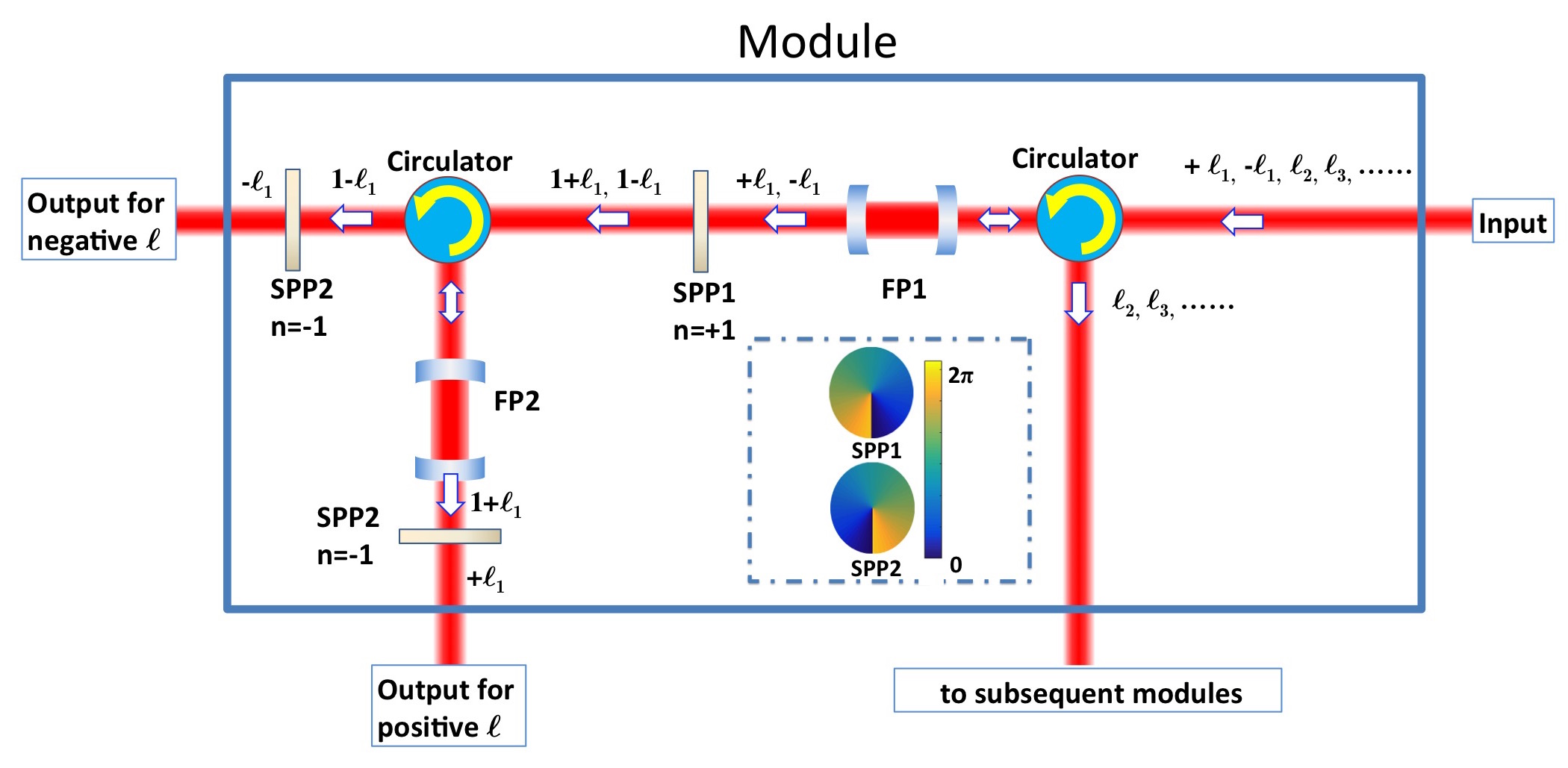


*Figure S5: Alternative design of the module that breaks the sign degeneracy of the OAM states. Two output ports instead of one are now used for the topological charges with opposite signs.*

**Section E—Efficiency of the OAM sorting method**

While FPs can be theoretically 100% efficient, in practice they are limited by the surface roughness of the mirror coatings/surfaces, alignment errors, and other imperfections. For a high finesse cavity such those used in cavity quantum electrodynamics (QED) experiments, scattering and absorption losses from the mirrors are the major source of loss^2^. A Thorlabs power meter was used to measure the incident continuous wave laser power entering and exiting the cavity at resonance. A peak transmitted power of 21.7% was measured for a mode-matched Gaussian beam, which is comparable with the majority of published alternative OAM sorting methods that report absolute efficiencies.

While a ~20% transmission is acceptable in the lab, higher transmissions are required for practical applications. Moving from the visible (~632.8 nm) to the infrared to minimize surface scattering and material absorption losses is the simplest method of improving efficiency. Optimization of the FP cavity and mirrors would also increase efficiency, as would the use of dielectric mirrors. For example, a FP cavity operating at a wavelength of 1064 nm has previously been reported with a transmission efficiency of 99.14% ± 0.86% with finesse above 2000.^3^ Commercial etalons already used in Dense Wavelength Division Multiplexing devices also display efficiencies above 90% with insertion losses below 1.5 dB at 1550 nm.^4^

For higher finesse cavities than the one presented here, however, additional considerations would represent additional limitations on the switching speed of the device. The previously-mentioned FP cavity with a finesse above 2000 was measured to take 958 ns for the transmitted intensity to decay to 1/e of its peak value. This cavity would therefore be limited by the photon lifetime within the cavity rather than the time taken for the cavity length to be switched to a different mode order.

Depending on the application, a trade-off between finesse and resolution would therefore be required. For OAM-multiplexing applications, for example, a cavity with finesse around the currently-used FP cavity could be selected to maximize transmitted power and switching speed.

1. Freise, A., Brown, D. & Bond, C. Finesse, frequency domain INterferomEter simulation softwarE. *arXiv preprint arXiv*:1306.2973 (2013).

2. Hood, C. J., Kimble, H. J., & Ye, J. Characterization of high-finesse mirrors: Loss, phase shifts, and mode structure in an optical cavity. *Physical Review A* **64**, 033804 (2001).

3. Sekiguchi H, *et al.* Ultralow-loss mirror of the parts-in-10 6 level at 1064 nm. *Optics Letters* **20**, 530-532 (1995).

4. Lightwaves2020, Lightwaves2020, Ed. 2015. Available from: [*www.Lightwaves2020.com*](http://www.Lightwaves2020.com)
